# Dynamic Control of Auxin Distribution Imposes a Bilateral-to-Radial Symmetry Switch during Gynoecium Development

**DOI:** 10.1016/j.cub.2015.02.011

**Published:** 2015-03-02

**Authors:** Laila Moubayidin, Lars Østergaard

(Current Biology *24*, 2743–2748; November 17, 2014)

In this article, we mistakenly referred to the *DR5::GFP* gynoecia in Figures 2A and 2B as developmental stage 5 in the legend as well as in the text. We should have referred instead to stage 7. It follows that “stage 5” in the model in Figure 4 should refer instead to “stage 7.” In addition, we have reevaluated the stages of some of the gynoecia in Figure S2 and have made corresponding corrections to the Figure S2 legend. The article and the Supplemental Information available online have been updated to reflect the correct labeling. None of the conclusions of our article are affected by these errors; however, the authors sincerely apologize for any confusion the errors may have caused.

