# Supplementary material for: Dynamic Control of Auxin Distribution Imposes a Bilateral-to-Radial Symmetry Switch during Gynoecium Development
Source: Curr Biol. 2014 Nov 17;24(22):2743–8. doi: 10.1016/j.cub.2014.09.080 (PMC4245708; doi:10.1016/j.cub.2014.09.080)
Supplement: Document S1. Supplemental Experimental Procedures and Figures S1–S3 [file mmc1.pdf]

Current Biology, Volume *24*

Supplemental Information

Dynamic Control of Auxin Distribution Imposes a Bilateral-to-Radial Symmetry Switch during Gynoecium Development

Laila Moubayidin and Lars Østergaard

Supplemental Figures

Figure S1.

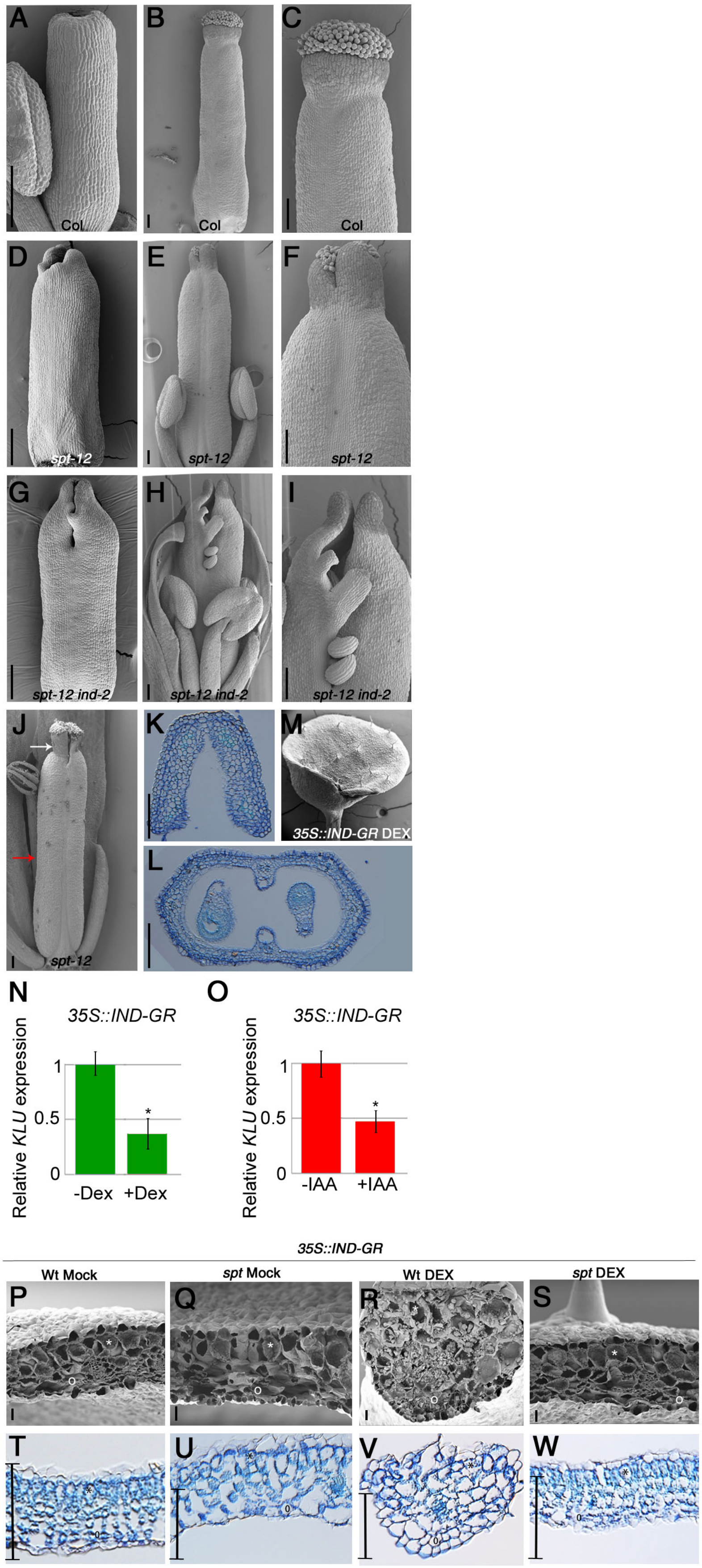

Figure S1. SPT and IND activities are both necessary and sufficient to govern organ radiality, Related to Figure 1.

(A-C) SEM images of gynoecia from Col-0 at stages 8 (A) and 10 (B,C). (D-F) SEM images of gynoecia from *spt-12* at stages 8 (D) and 10 (E,F). (G-I) SEM images of gynoecia from *ind-2 spt-12* at stages 8 (G) and 10 (H,I). (J) SEM of *spt-12* gynoecium at stage 12. White arrow indicates the style region, red arrow indicates the ovary. (K,L) Toluidine blue-staining of cross sections from *spt-12* stage-12 gynoecium in the style (K) and ovary (L). (M) SEM image of radicalized, cup-shaped seedling from *35S::IND:GR* on 10µM DEX. Scale bars in (A-M) represent 100µm.

(N) qRT-PCR of *KLU* in *35S::IND:GR* with 10µM DEX. (O) qRT-PCR of *KLU* with 50µM IAA. Error bars show SD. Student's t-test, *$p<0.05$. (P-W) SEM images (P-S) and toluidine blue-stained (T-W) of sections of rosette leaf from *35S::IND:GR* in Col-0 (P,T) and *spt-12* (Q,U) without DEX and *35S::IND:GR* in Col-0 (R,V) and *spt-12* (S,W) with 10µM DEX. Asterisks indicate the palisade parenchyma, circles indicate sponge parenchyma. Note that radialized leaves emerging after IND overexpression appear not to show changing in the identity of the canonical internal leaf tissues. Scale bars in (P-S) represent 10µm, in (Q-T) represent 100µm.

Figure S2.

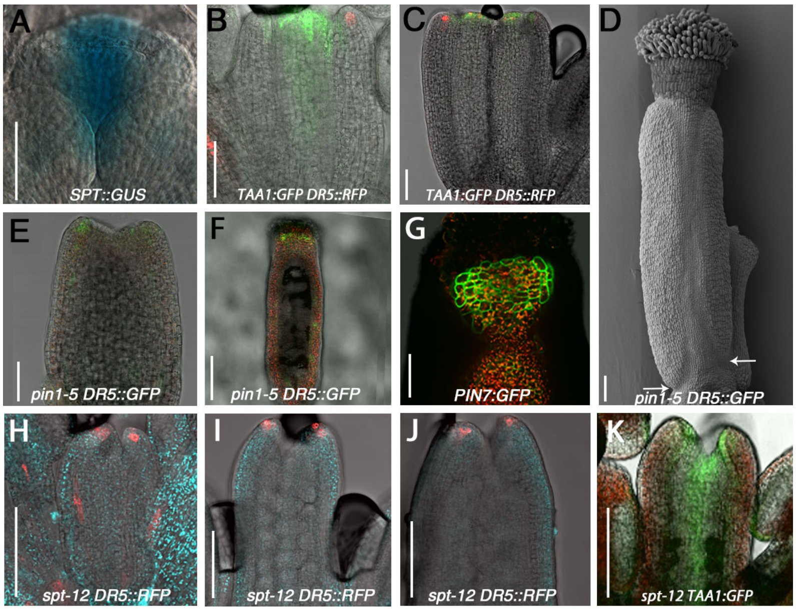

Figure S2. Lateral and medial auxin-signaling foci drive apical-basal growth and style formation, respectively, during gynoecium development, Related to Figure 2.

(A) *SPT::GUS* in the medial region of a stage-5 Col-0 gynoecium. Scale bar represents 50µm. (B-C) Confocal image of *TAA1::TAA1:GFP* and *DR5::RFP* of a stage-6 (B) and a stage-9 (C) Col-0 gynoecium. *TAA1::TAA1:GFP* and *DR5::RFP* are expressed in complementary regions during early developmental stages since *TAA1::TAA1:GF*P expression is constrained in the medial region while *DR5::RFP* shows expression in the lateral (B). At stage-9 *TAA1::TAA1:GFP* and *DR5::RFP* expression overlaps since *TAA1::TAA1:GFP* starts to be expressed in the top adaxial side of the lateral region while *DR5::RFP* is expressed in the top medial foci. Scale bar represents 50µm. (D-F) SEM image (D) and confocal analysis (E,F) of *pin1-5 DR5::GFP* gynoecium at stage 11 (D), stage 7 (E) and 10 (F). White arrows in (C) indicate the base of the ovary. Scale bars represent in (D) 100µm and in (E,F) 50µm. (G) *PIN7::PIN7:GFP* in stage-10 Col-0 gynoecium. Scale bar

represents 50µm. (H-J) *DR5::RFP* in *spt-12* at stages 5-6 (F), 8-9 (G) and 10 (H). Note the absence of the medial *DR5* signaling foci over developmental stages. Scale bars represent 100µm. (K) *TAA1::TAA1:GFP* expression in *spt-12* at stage 5-6. Scale bar represents 100 µm.

Figure S3.

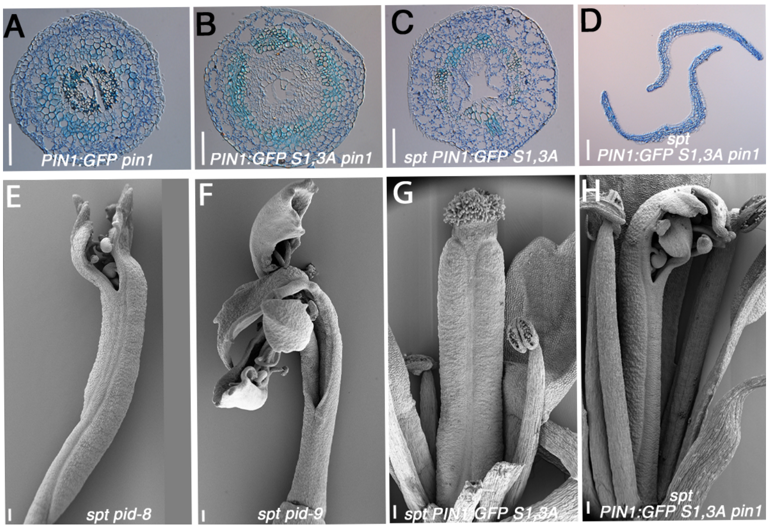

Figure S3. Radial style is formed by the coordinated activity of lateral and medial auxin foci, Related to Figure 3.

(A-D) Toluidine blue-stained style sections in *PIN1::PIN1:GFP pin1* (A), *PIN1::PIN1:GFP S1,3A pin1* (B), *PIN1::PIN1:GFP S1,3A spt-12* (C) and *PIN1::PIN1:GFP S1,3A spt-12 pin1* (D). Scale bars represent 100µm. (E-H) SEM images of stage-10 gynoecia from *pid-8 spt-12* (E), *pid-9 spt-12* (F), *PIN1::PIN1:GFP S1,3A spt-12* (G), and *PIN1::PIN1:GFP S1,3A spt-12 pin1* (H). Scale bars in (A-D) represent 100µm.

Supplemental Experimental Procedures

Plant Materials and Growth Conditions

Plants were grown on soil in long days condition (16h light/8h dark) in the glasshouse. Mutant lines *ind-2 spt-12* [15], *spt-12* [15,S1], *35S::IND-GR* and *35S::IND-GR spt-12* [15,16], *pid-9* [20], *PIN1::PIN1:GFP pin1*, *PIN1::PIN1:GFP S1,3A pin1* and *PIN1::PIN1:GFP S1,2,3E pin1* [22] were in Col-0 background. *pid-8* [23] was in Ws background. Reporter lines of *SPT::GUS* [14,S2], *KLU::GUS* [11], *PID::GUS* [19], *TAA1::TAA1:GFP* [17], *DR5::GFP* [S3], *DR5::RFP* [S4], *pin1 DR5::GFP* [S5], *PIN1::PIN1:GFP* [S6], *PIN3::PIN3:GFP* [S7], *PIN7::PIN7:GFP* [28].

RNA extraction and qRT-PCR

Total RNA was isolated from Col-0, *spt-12* and *spt-12 ind-2* inflorescences and from *35S::IND-GR* seedlings treated or untreated with DEX or IAA as shown in [16], and treated with RNeasy Plant Mini Kit (QIAGEN). Each RNA sample was reverse transcribed using the M-MLV Reverse Transcriptase (Promega) according to the manufacturer's instructions. qRT-PCR was performed in triplicates from each RNA sample and repeated twice using BRYT Green based GoTaq qPCR Master Mix (Promega) with Chromo4 Real-Time PCR Detection System (Bio-Rad). Expression levels were calculated relative to *UBIQUITIN 10* using the $2^{-\Delta\Delta ct}$ method. Statistical analysis was done in MS Exel (ANOVA: Single Factor) using $p<0.05$. Primers were designed according to the recommendations of Applied Biosystems Quantitative RT-PCR (qRT-PCR) analysis was conducted using the gene-specific primers listed below:

for *KLU*:

KLU FWD: AGGCTGGTGAGTGAAGGCTA

KLU REV: CAAGCCAAGCAAGACATCAA

for *UBIQUITIN 10*:

UB10 FWD: AGAACTCTTGCTGACTACAATATCCAG

UB10 REV: GTTAAGACGTTGACTGGGAAAACTAT

GUS histochemical assay

To visualize *KLU::GUS*, *PID::GUS* and *SPT::GUS* lines, GUS histochemical assay was performed using 1 mg/ml of ß-glucoronidase substrate X-gluc (5-bromo-4-chloro-3-indolyl glucuronide, MELFORD) dissolved in Dimethyl sulfoxide (DMSO). X-Gluc solution contains 100 mM sodium phosphate buffer, 10mM EDTA, 0.5 mM K_3 $Fe(CN)_6$, 3 mM $K_4Fe(CN)_6$, 0.1% Triton X100 according to the JIC standard operating procedures. Wild type and mutant inflorescences of *KLU::GUS* and *PID::GUS* were vacuum infiltrated for 10' and incubated for 16 hours at 37°C in the dark. *SPT::GUS* inflorescences were pre-treated for 1h with acetone at -20°C, washed two times for 5' in 100 mM sodium phosphate buffer, washed for 30' in 100 mM sodium phosphate buffer containing 1mM K_3 - K_4 at room temperature and then incubated for 2h at 37°C in the X-Gluc solution. After staining, the reaction buffer was replaced with 70% ethanol until chlorophyll was completely washed out from the samples. Gynoecium were dissected, mounted in Chlorohydrate (Sigma) solution and analyzed using Leica DM600 light microscopy. Images were taken using Leica LAS AF7000 software.

Toluidine Blue Staining

Tissues were fixed for 16h at 25°C in 3.7% formaldehyde, 5% acetic acid, and 50% ethanol and subsequently dehydrated through an ethanol series until 70%. The tissues were embedded in paraffin. An RM 2125 rotary microtome (Leica) was used to make 10 mm transverse sections of Col, *spt-12* and *spt-12 ind-2* gynoecium at stage 12, and *35S::IND-GR* and *spt-12 35S::IND-GR* leaves treated with either mock or DEX. Sections were deparaffinizated by two rounds of incubation in 100% Histoclear (National Diagnostics) for 10' at room temperature followed by two washes in 100% ethanol for 2' at room temperature, air dried for 30' and stained for 10' by an aqueous solution containing 0.005% Toluidine blue O (ACROS ORGANICS). Slides were washed for 1' in water; sections were mounted in a histological mounting medium Histomount (National Diagnostic) and examined under Leica DM600 light microscopy. Images were taken using Leica LAS AF7000 software.

Scanning Electron Microscopy

Different stages of Col, *spt-12* and *spt-12 ind-2* gynoecium and seedlings of *35S::IND-GR* and *spt-12 35S::IND-GR* leaves treated with either mock or DEX were fixed 16h at 25°C in 3.7% formaldehyde, 5% glacial acetic acid, and 50% ethanol. After a complete dehydration through an ethanol series until 100%, gynoecium and leaves were critical point dried. For the sections of *35S::IND-GR* and *spt-12 35S::IND-GR* leaves in Figure 1 and S1, only the first leaves were used and cut before the critical point drying. Samples were dissected and coated with gold and examined under Zeiss Supra 55VP Field Emission Scanning Electron Microscope using an acceleration voltage of 3 kV.

DEX and IAA inductions

For *IND* ectopic induction, seeds carrying the *35S::IND-GR* construct, in wild type and in *spt-12* background, were surface sterilized using 50% bleach for 10 minutes and then rinsed four times with sterile water. After 5 days of cold treatment, *A. thaliana* seeds were plated and grown for two weeks, in a horizontal position, at 22°C in long-day conditions (16 hours light/8 hours dark cycle) on MS (Murashige & Skoog) medium containing micro and macro elements including vitamins (Formedium Limited), 3% sucrose at pH 5.8, supplemented with 10 μM dexamethasone (Dex, Sigma-Aldrich), prepared from a 10 mM stock in ethanol, or an equivalent amount of ethanol, as mock treatment. For qRT-PCR experiment with *35S::IND-GR* construct, seedlings were treated as previously shown in [16].

Confocal Microscopy

Confocal microscopy was performed using a Leica SP5 laser scanning microscope equipped with an Argon krypton laser (Leica Microsystems). The 488-nm and 561-nm excitation line of an argon ion laser was used to excite GFP and RFP, respectively. GFP emission spectra were collected between 497 and 551 nm, RFP emission spectra were collected between 570 and 630 nm, and plastid autofluorescence was collected between 624 and 699nm. For the lateral view of gynoecium in Figures 2A, 2C, 2E, 2G, 2I, 2J, 2M-2R and S2B, S2C, S2E-S2J

floral buds were dissected, mounted in water and observed using transmitted light (bright field) using x10 air or x40 oil objectives. For the top views of style region in Figures 2B,D,F,H,K,L and 3D,E,G gynoecium were dissected and mounted vertically in an agars dishes, we used the X25/0.95 water dipping objective lens that allowed a three dimensional visualization of the specimens. Images were averaged 8 times and were processed using the Leica CONFOCAL software. For Figures 2M-2O, 3I and Movie1, 2-3μm Z sections were imaged and, for the figures, converted to 3D projections.

Supplemental References

[S1] Ichihashi, Y., Horiguchi, G., Gleissberg, S., and Tsukaya, H. (2010). The bHLH transcription factor SPATULA controls final leaf size in *Arabidopsis thaliana*. Plant Cell Physiol *51*, 252-261.

[S2] Groszmann, M., Bylstra, Y., Lampugnani, E.R., and Smyth D.R. (2010). Regulation of tissue-specific expression of SPATULA, a bHLH gene involved in carpel development, seedling germination, and lateral organ growth in *Arabidopsis*. J Exp Bot *61*, 1495-1508.

[S3] Ottenschläger, I., Wolff, P., Wolverton, C., Bhalerao, R.P., Sandberg, G., Ishikawa, H., Evans, M., and Palme, K. (2003). Gravity-regulated differential auxin transport from columella to lateral root cap cells. Proc. Natl Acad. Sci. USA *100*, 2987–2991.

[S4] Marin, E., Jouannet, V., Herz, A., Lokerse, A.S., Weijers, D., Vaucheret, H., Nussaume, L., Crespi, M.D., and Maizel, A. (2010). miR390, Arabidopsis TAS3 tasiRNAs, and their AUXIN RESPONSE FACTOR targets define an autoregulatory network quantitatively regulating lateral root growth. Plant Cell *22*, 1104–1117.

[S5] Ružicka, K., Ljung, K., Vanneste, S., Podhorská, R., Beeckman, T., Friml, J. and Benková, E. (2007). Ethylene regulates root growth through effect on auxin biosynthesis and transport-dependent auxin distribution. Plant Cell *19*, 2197-2212.

[S6] Benková, E., Michniewicz,M., Sauer, M., Teichmann, T., Seifertová D., Jurgens, J., and Friml, J. (2003). Local, Efflux-Dependent Auxin Gradients as a Common Module for Plant Organ Formation. Cell *115*, 591-602.

[S7] Žádníková, P., Petrášek, J., Marhavý, P., Raz, V., Vandenbussche, F, Ding, Z., Schwarzerová, K., Morita, M.T., Tasaka, M., Hejátko, J., Van Der Straeten, D., Friml, J., and Benková, E. (2010). Role of PIN-mediated auxin efflux in apical hook development of Arabidopsis thaliana. Development *137*, 607-617.
