# Supplementary material for: Dynamic Control of Auxin Distribution Imposes a Bilateral-to-Radial Symmetry Switch during Gynoecium Development
Source: Curr Biol. 2014 Nov 17;24(22):2743–8. doi: 10.1016/j.cub.2014.09.080 (PMC4245708; doi:10.1016/j.cub.2014.09.080)
Supplement: Document S2. Article plus Supplemental Information [file mmc3.pdf]

Current Biology 24, 2743–2748, November 17, 2014 ©2014 The Authors http://dx.doi.org/10.1016/j.cub.2014.09.080

Report

Dynamic Control of Auxin Distribution Imposes a Bilateral-to-Radial Symmetry Switch during Gynoecium Development

Laila Moubayidin[1] and Lars Østergaard[1,*]
[1]Department of Crop Genetics, John Innes Centre, Norwich Research Park, Norwich NR4 7UH, UK

Summary

Symmetry formation is a remarkable feature of biological life forms associated with evolutionary advantages and often with great beauty. Several examples exist in which organisms undergo a transition in symmetry during development [1–4]. Such transitions are almost exclusively in the direction from radial to bilateral symmetry [5–8]. Here, we describe the dynamics of symmetry establishment during development of the *Arabidopsis* gynoecium. We show that the apical style region undergoes an unusual transition from a bilaterally symmetric stage ingrained in the gynoecium due to its evolutionary origin to a radially symmetric structure. We also identify two transcription factors, INDEHISCENT [9] and SPATULA [10], that are both necessary and sufficient for the radialization process. Our work furthermore shows that these two transcription factors control style symmetry by directly regulating auxin distribution. Establishment of specific auxin-signaling foci and the subsequent development of a radially symmetric auxin ring at the style are required for the transition to radial symmetry, because genetic manipulations of auxin transport can either cause loss of radialization in a wild-type background or rescue mutants with radialization defects. Whereas many examples have described how auxin provides polarity and specific identity to cells in a range of developmental contexts, our data presented here demonstrate that auxin can also be recruited to impose uniform identity to a group of cells that are otherwise differentially programmed.

Results and Discussion

Transition to Radial Symmetry at the *Arabidopsis* Gynoecium Apex Occurs through Repression of Margin Identity

Symmetry transitions are common during embryogenesis of all multicellular organisms [1–4]. In most cases, the transition is from radial to bilateral symmetry and controlled by *Hox* and *decapentaplegic* genes in animals [5, 6]. In fact, the echinoderms provide the only reported example in which this order is reversed such that the radially symmetric animal develops from a bilaterally symmetric larvae stage [7, 8].

In the model plant *Arabidopsis thaliana*, the gynoecium is derived from the fusion of two carpels and forms in the center of the flower. During gynoecium development, the apical style becomes radially symmetric with stigmatic papillae arising [11] (Figure 1A and Figures S1A–S1C available online), suggesting the existence of a switch from bilateral to radial symmetry.

Given that the *Arabidopsis* gynoecium originates from two fused leaves [11, 12], it is likely that factors involved in specifying leaf margin tissue are also regulated in the gynoecium. Although margin identity genes may have a role in defining margins in the bilaterally symmetric ovary, we would expect such activities to be repressed in the style to achieve radial symmetry. *KLUH* (*KLU*) is a margin-identity gene expressed in peripheral cells of *Arabidopsis* petals and in the marginal tissue of the gynoecium [13]. Expression of *KLU*::*GUS* was detected along the entire length of developing gynoecia at stage 9 (Figure 1B) but lost at the style of the mature gynoecium (stage 12 in Figure 1C; developmental stages defined in [14]).

Mutations in the *SPATULA* (*SPT*) gene lead to a failure in radial symmetry establishment at the style [10] (Figures 1D and S1D–S1F). Interestingly, in the *spt-12* mutant, *KLU*::*GUS* was still expressed in the apical medial part throughout gynoecium development (Figure 1E). These results suggest that the bilateral-to-radial transition occurring during style formation requires transcriptional repression of margin-identity genes.

INDEHISCENT and SPATULA Impose Organ Radialization

When the *spt* mutant is combined with mutations in the *INDEHISCENT* (*IND*) gene [9], the effect on style and stigma development is significantly enhanced reflecting the synergistic activities of these two basic helix-loop-helix transcription factors (Figures 1J and S1G–S1I) [15]. In the wild-type gynoecium, the ovary has a bilateral symmetry plane in which the septum divides the ovary into two separate locules, whereas the style is a rounded, compact, and radially symmetric structure (Figures 1G–1I). *spt* and *ind spt* have defects in septum formation but maintain bilateral symmetry in the ovary (Figures 1J, 1L, S1J, and S1L). The style in these mutants fails to acquire radial symmetry showing that IND and SPT are required to ensure radial symmetry establishment at the gynoecium apex (Figures 1K and S1K). *KLU* expression was found to be significantly upregulated in *spt* and *ind spt* mutants (Figure 1F) and downregulated in a *35S*::*IND*:*GR* line [16] induced by dexamethasone (DEX) (Figure S1N). This is in agreement with a role of IND and SPT in promoting radial symmetry, at least partially, by repressing margin identity.

We next tested whether IND and SPT are sufficient to establish radial symmetry in an alternative developmental context such as a bilaterally symmetric flat leaf. To this end, the DEX-inducible *35S*::*IND*:*GR* line was grown on medium supplemented with DEX. After 15 days, completely radialized leaves emerged as rod-like and cup-like structures (Figures 1M, 1O, and S1M). Notably, the epidermal cell shape of these radialized leaves is reminiscent of the shape of style cells (Figure 1S and inset 1S'), which is in contrast to the normal jigsaw-shaped leaf epidermal cells from noninduced plants (Figure 1Q). Conversely, anatomical analyses of the internal cell types in cross-sections suggest that *IND* overexpression reprograms only the marginal cells (Figures S1P, S1R, S1T, and S1V). The IND-driven organ radialization was completely dependent on the presence of SPT function, because the effect was lost in the *spt-12* mutant background (Figures 1N, 1P, 1R, 1T, S1Q, S1S, S1U, and S1W). Altogether, these results

*Correspondence: lars.ostergaard@jic.ac.uk
This is an open access article under the CC BY license (http://creativecommons.org/licenses/by/3.0/).

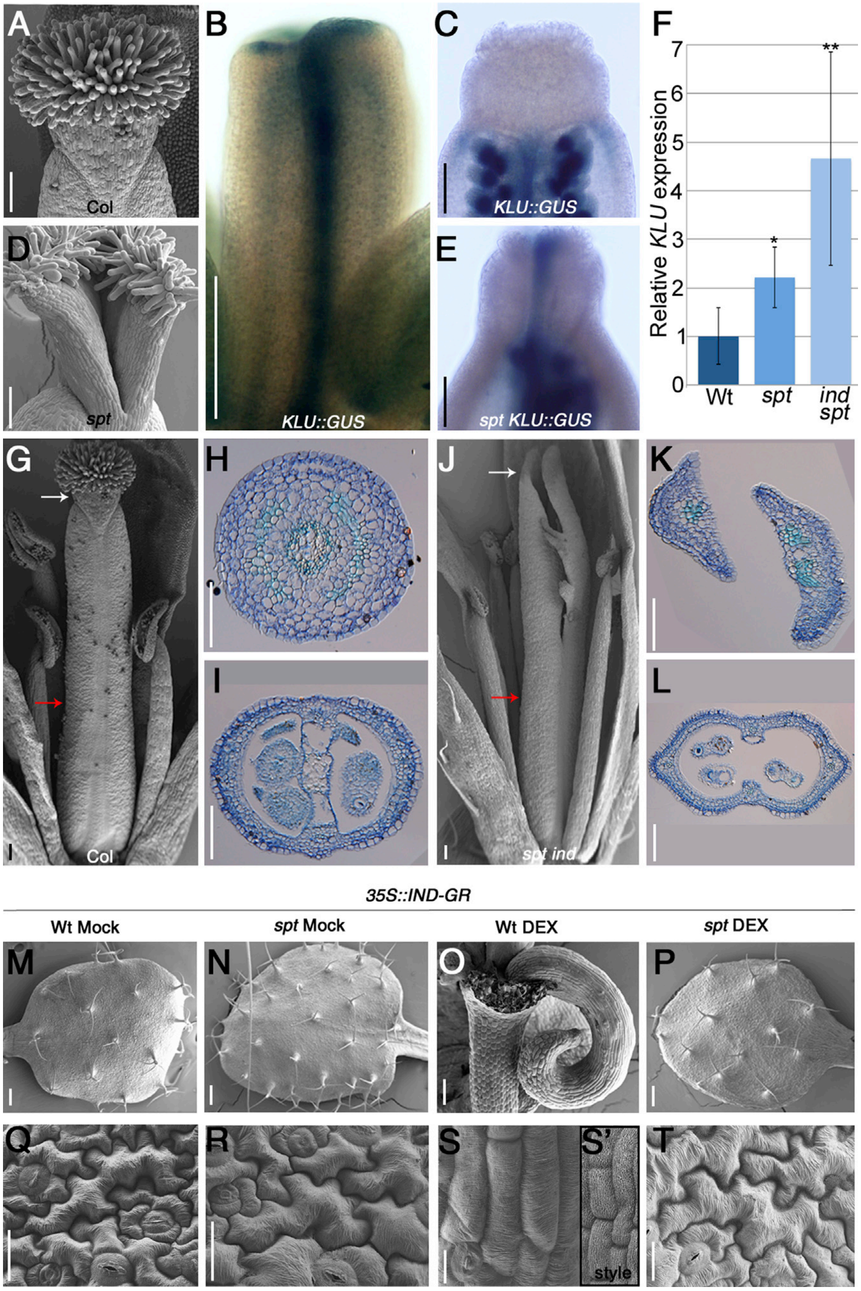

Figure 1. Radial Symmetry in the *Arabidopsis* Gynoecium Is Imposed by the Activities of *IND* and *SPT*

(A) SEM image of the apical region of wild-type Col-0 gynoecia at stage 13.

(B and C) *KLU*::*GUS* in Col-0 at stage 9 (B) and stage 12 (C).

(D) SEM image of the apical region of *spt-12* gynoecium at stage 13.

(E) *KLU*::*GUS* in *spt-12* at stage 12. The scale bars in (A)–(E) represent 100 μm.

(F) *KLU* quantitative RT-PCR in Col-0, *spt-12*, and *ind-2 spt-12*. Error bars show SDs. Student's t test; *p < 0.05; **p < 0.01. WT, wild-type.

(G–I) Col-0 gynoecia at stage 13. SEM (G) and Toluidine blue-stained cross-sections of the style (H) and ovary (I).

(J–L) *ind-2 spt-12* double-mutant gynoecium at stage 13. SEM (J) and Toluidine blue-stained cross-sections of the style (K) and ovary (L). In (G) and (J), white arrow indicates the style region and red arrow indicates the ovary. The scale bars in (G)–(L) represent 100 μm.

(M and N) SEM images of rosette leaf from *35S*::*IND*:*GR* in Col-0 (M) and *spt-12* (N) without DEX.

(O and P) *35S*::*IND*:*GR* in Col-0 (O) and *spt-12* (P) with 10 μM DEX. The scale bars in (M)–(P) represent 200 μm.

(Q–T) SEM of rosette leaf epidermal cells from genotypes and treatments depicted in (M)–(P). Note that induction of IND imposes a change from jigsaw-shaped leaf epidermal cells to cylindrical-shaped cells resembling wild-type style cells in the inset (S'). The scale bars in (Q)–(T) represent 20 μm.

See also Figure S1.

show that both IND and SPT are necessary and sufficient for mediating organ radialization.

Auxin Transport and Signaling Is Dynamic during Gynoecium Growth

During gynoecium development, auxin distribution is tightly controlled in both time and space. Two apical foci of the auxin-signaling reporter, *DR5*::*GFP*, are established in the lateral apical domains at early stages (5/6) of organ development (Figures 2A and 2B) [17]. Subsequently, two medial foci emerge at stage 8/9 (Figures 2C and 2D; Movie S1), and immediately prior to formation of the style (stage 10), all four foci are connected in an auxin ring of radial symmetry (Figures 2E and 2F). This pattern mimics the transition of bilateral-to-radial symmetry suggesting a role for the spatiotemporal dynamics of auxin in symmetry establishment.

We initially tested if the auxin-signaling foci are established by local auxin production. The *TRYPTOPHANE AMINOTRANSFERASE OF ARABIDOPSIS1* (*TAA1*) gene encodes an auxin-biosynthesis enzyme and is expressed in the same region as SPT during early stages of gynoecium development (Figures S2A and S2B) [18]. *TAA1* and its closest homolog *TAR2* likely regulate auxin dynamics in the gynoecium, because the *taa1 tar2* double mutant exhibits a split-style phenotype [18]. We conducted the expression analysis of a *TAA1*::*TAA1*:*GFP* line concomitantly with *DR5*::*RFP* to correlate the dynamics of auxin production and auxin signaling in vivo. Early in development, expression of these two reporters is nonoverlapping with *DR5*::*RFP* in the apical lateral part and *TAA1*::*TAA1*:*GFP* in the medial region (Figure S2B). At stage 9, there is overlap in the medial region with *TAA1*::*TAA1*:*GFP* expanding to the lateral adaxial side (Figure S2C). Because the *DR5*::*RFP* signal in the lateral foci appears before the *TAA1*::*TAA1*:*GFP* signal, it is unlikely that the two lateral auxin-signaling foci are established by local auxin synthesis.

Next, we analyzed if auxin transport is involved in establishing the auxin-signaling foci. The *PIN1* gene encodes a plasma membrane (PM) localized member of the PIN auxin efflux family that directs polar auxin transport (PAT) via their asymmetric subcellular localization [19, 20]. PIN1 protein is located apically

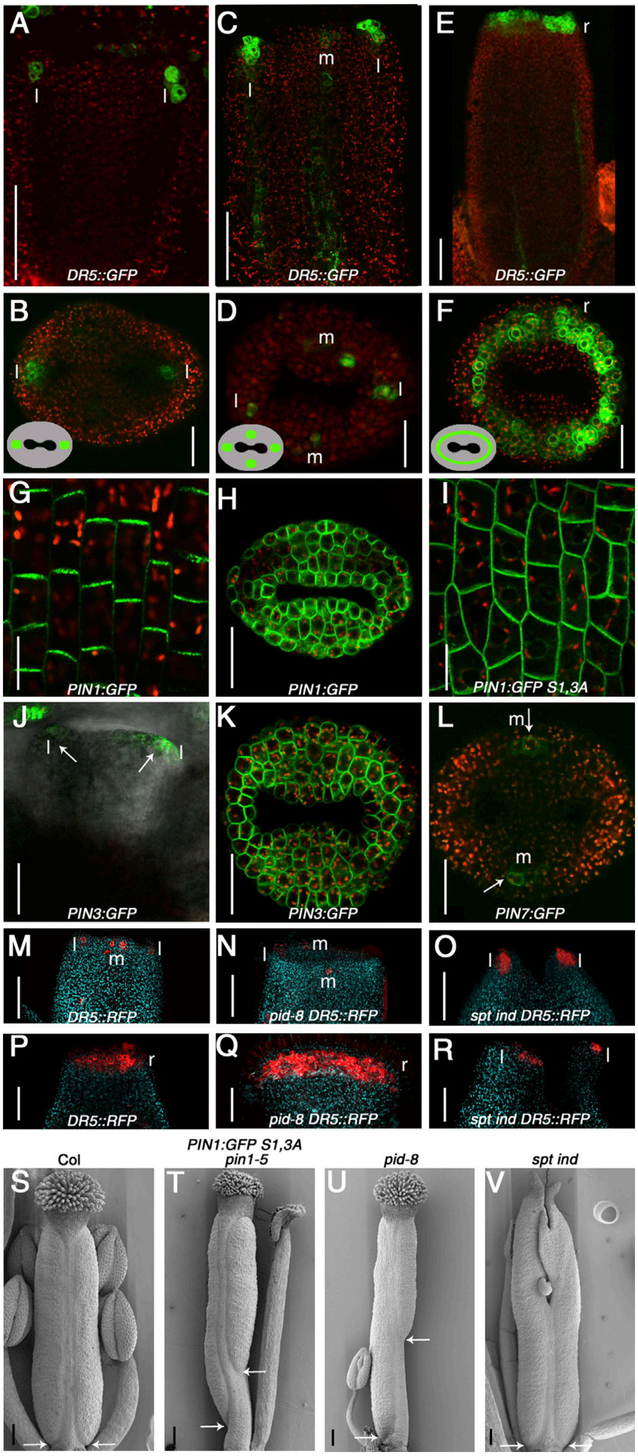

Figure 2. Auxin Is Dynamically Distributed at the Apex of the Developing Gynoecium and Functions in Sustaining Apical-Basal Growth and Establishing Radial Symmetry

(A–F) Confocal images of *DR5*::*GFP* in Col-0 at stage 5 (A and B), stage 8 (C and D), and stage 10 (E and F). Upper images are longitudinal views (A, C, and E), and lower images are top views (B, D, and F). l indicates the position of the lateral auxin foci, and m indicates the position of the medial auxin foci. Insets in (B), (D), and (F) indicate the position of the GFP signal in the outline of the gynoecium viewed from the top. The scale bars in (A), (C), and (E) represent 50 μm and in (B), (D), and (F) represent 25 μm.

(G) *PIN1*::*PIN1*:*GFP* stage 9 showing ovary expression in medial region and apical localization presumably transporting auxin toward the top. The scale bar represents 10 μm.

(H) *PIN1*::*PIN1*:*GFP* stage 8 showing strongest expression in medial style region and apolar localization of the PIN1:GFP protein (top view). The scale bar represents 25 μm.

(I) PIN1 apolar localization in ovary of *PIN1*::*PIN1*:*GFP S1,3A pin1-5* at stage 9. The scale bar represents 10 μm.

(J) Lateral view of *PIN3*::*PIN3*:*GFP* stage 5 with expression in lateral foci (arrows).

(K) Top view of *PIN3*::*PIN3*:*GFP* stage 9 showing expansion of expression in a ring at the position of the presumptive style and apolar localization.

(L) Top view of *PIN7*::*PIN7*:*GFP* stage 7 showing expression in the medial foci (arrows). The scale bars in (J)–(L) represent 25 μm.

(M–R) Confocal images of *DR5*::*RFP* at stage 8 (M–O) and stage 10 (P–R) in Col-0 (M and P), *pid-8* (N and Q), and *ind-2 spt-12* (O and R). The scale bars in (M)–(R) represent 50 μm.

(S–V) SEM images of stage 11 gynoecia from Col-0 (S), *PIN1*::*PIN1*:*GFP S1,3A pin1-5* (T), *pid-8* (U), and *ind-2 spt-12* (V). White arrows indicate the base of the ovary. The scale bars in (S)–(V) represent 100 μm.

See also Figure S2.

in cells of the ovary presumably to direct auxin flux from the base to the top of the developing gynoecium [16] (Figure 2G). At the apex, PIN1 localization becomes apolar primarily in the medial part of the gynoecium (Figure 2H). PIN1-mediated auxin transport is therefore likely to contribute to the specific pattern of auxin distribution at the apex. Indeed, in gynoecia from a weak *pin1* mutant allele (*pin1-5*), the intensity of the two lateral *DR5*::*GFP* foci are severely reduced and apical-basal polarity defects are detected (Figures S2D–S2F). An identical effect occurs in plants with mutations in the *PINOID* (*PID*) gene encoding an AGC3-type protein kinase that promotes apical PIN localization at the PM by phosphorylating specific serine residues in PIN proteins [21–24] (Figures 2M, 2N, 2S, and 2U). Indeed, mutations in two of those specific serine residues (*PIN1*:*GFP S1,3A*) [24] lead to apolar distribution of PIN1 along the gynoecium (Figure 2I) and apical-basal growth defects similar to the weak *pid-8* mutant [25] (Figures 2T and 2U). Moreover, this growth-defective phenotype is reminiscent of treatment with the PAT inhibitor NPA [26, 27].

Another member of the PIN family, PIN3 is initially confined to a few laterally positioned apical cells (Figure 2J) overlapping with the lateral *DR5*::*GFP* foci (Figure 2A). Later, PIN3 is detected throughout the apex in the same domain as *DR5*::*GFP* (Figures 2F and 2K) with apolar localization of the protein (Figures 2J and 2K). A third PIN member, PIN7, is localized apolarly in a few medially positioned apical cells from around stage 7 (Figure 2L), presumably joining the activity of PIN1 in establishing the medial foci. At later stages, PIN7 is found throughout the apex sustaining the ring formation similarly to PIN3 (Figure S2G). Expression and localization of PIN1/PIN3/PIN7 suggests that PAT mediates the transition from a bilaterally to a radially distributed auxin response (Figures 2A, 2B, 2E, and 2F). The requirement for apolarly localized PINs to establish the radial auxin maximum at the gynoecium apex resembles the apolar localization of PIN4 around the quiescent center cells of the root apical meristem and its precursor cells during embryogenesis [28, 29]. In this tissue, PIN4 is necessary for the proper positioning of the auxin-response maximum at the embryo stem cell niche [29].

Lateral and Medial Auxin-Signaling Foci Control Gynoecium Symmetry

To address the role of the lateral and medial pairs of auxin-signaling foci, we tested *DR5* expression dynamics in mutants with defects in either apical-basal growth or style development. *DR5*::*GFP* in *pin1-5* and *DR5*::*RFP* in *pid-8* mutants

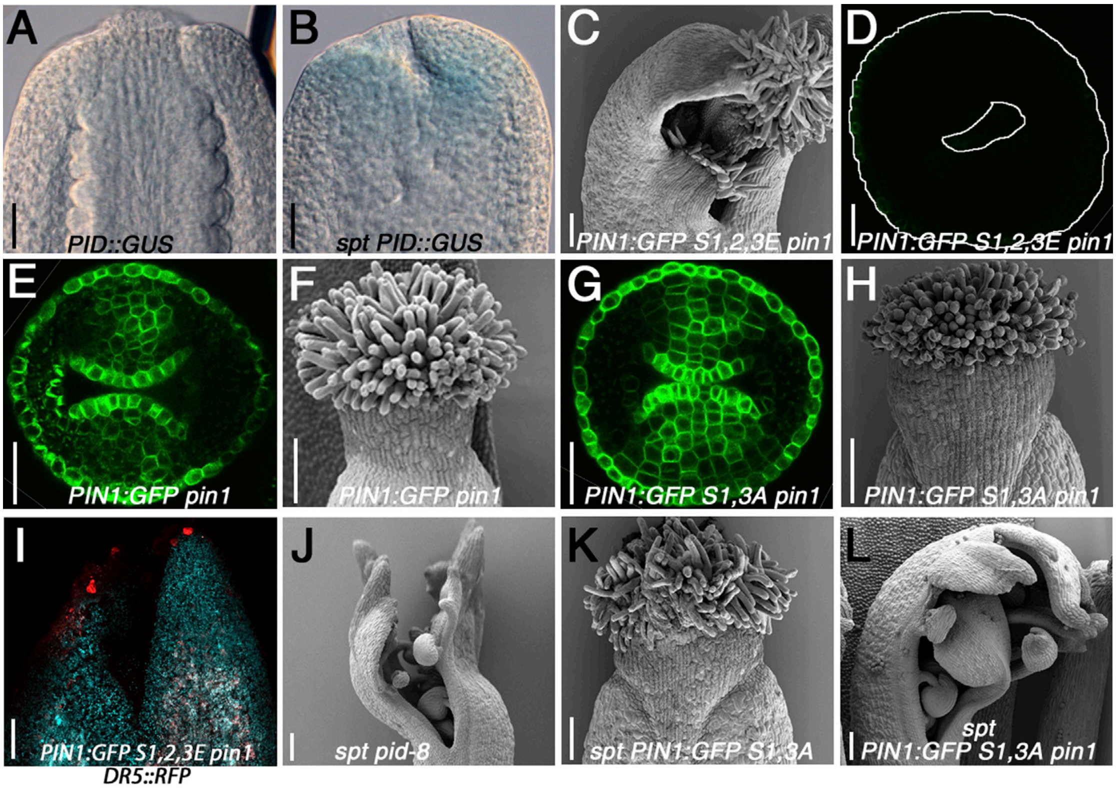

Figure 3. Control of PID-Directed PIN Phosphorylation Is Required for Radial Symmetry, and Induced Apolar Transport at the Style Is Sufficient to Rescue Mutants with Radial Defect

(A and B) *PID*::*GUS* expression in Col-0 (A) and *spt-12* (B) at stage 9. The scale bars represent 25 μm.
(C) SEM image of *PIN1*::*PIN1*:*GFP S1*,*2*,*3E pin1* at stage 13.
(D) Confocal top-view image of *PIN1*::*PIN1*:*GFP S1*,*2*,*3E pin1* at stage 8.
(E and F) *PIN1*::*PIN1*:*GFP pin1* with confocal top view at stage 8 (E) and SEM at stage 13 (F).
(G and H) *PIN1*::*PIN1*:*GFP S1*,*3A pin1-5* with confocal top view at stage 8 (G) and SEM at stage 13 (H). The scale bars in (C), (F), and (H) represent 100 μm and in (D), (E), and (G) represent 25 μm.
(I) Confocal images of *DR5*::*RFP* at stage 10 from *PIN1*::*PIN1*:*GFP S1*,*2*,*3E pin1*. The scale bar represents 50 μm.
(J–L) SEM of stage 10 gynoecia from *pid-8 spt-12* (J), *PIN1*::*PIN1*:*GFP S1*,*3A spt-12* (K), and *PIN1*::*PIN1*:*GFP S1*,*3A spt-12 pin1* (L). The scale bars in (J)–(L) represent 100 μm.
See also Figure S3.

showed a drastically decreased signal in the lateral foci, whereas the auxin ring appeared normally, thus correlating with radial style formation (Figures 2M, 2N, 2P, 2Q, 2U, and S2D–S2F). As in many organ-development processes, gynoecium growth along the apical-basal polarity axis follows the direction of auxin flux, directing growth toward the two lateral auxin foci providing cell and tissue polarity [30]. In agreement with the reduced lateral *DR5* signals, *pin1-5* and *pid-8* mutants show apical-basal growth defects (Figures 2U and S2D). Therefore, the two lateral foci are important to ensure apical-basal growth of the two carpels.

In mutants with defects in the bilateral-to-radial symmetry transition, the two lateral *DR5* foci are correctly established early during gynoecium development, and these mutants have no apparent apical-basal defects (Figures 2V, S1J, and S2H). In contrast, the medial *DR5* foci were not established in these mutant backgrounds (Figures 2O and S2I) and the *DR5* ring fails to form (Figures 2R and S2J) [15]. The lack of *DR5*::*RFP* in *spt-12* is unlikely to be due to lack of auxin biosynthesis, because *TAA1*::*TAA1*:*GFP* is still expressed in *spt-12* (Figure S2K). These results suggest that the medial auxin-signaling foci promote the bilateral-to-radial symmetry switch. In agreement with this, the medial *DR5* foci form normally in *pid-8* gynoecia with no defect in establishing the *DR5* ring and correlating with formation of a radial style (Figures 2N and 2Q).

Disrupting Apolar PIN1-Mediated Auxin Distribution at the Gynoecium Apex Abolishes Radial Symmetry Transition

It was previously shown that SPT and IND directly repress *PID* expression [15, 16]. Accordingly, we found that a *PID*::*GUS* reporter was ectopically expressed in the style region of the *spt-12* mutant compared to wild-type (Figures 3A and 3B). The importance of apolar PIN1 localization was analyzed by expressing a version of PIN1 that mimics constitutive phosphorylation of the three serine residues targeted by PID (*PIN1*:*GFP S1*,*2*,*3E*) in the *pin1* mutant background [24]. Gynoecia from this line exhibited a split-style phenotype similar to the *spt-12* mutant (Figures 1D, 3C, and 3F). Interestingly PIN1:GFP S1,2,3E protein could not be detected at the apex as opposed to a nonmutated PIN1:GFP version (Figures 3D and 3E), suggesting that apical localization renders PIN1 unstable in this tissue. Consistent with defective PIN1:GFP S1,2,3E protein localization, *DR5*::*RFP* was not detected in the medial foci of *PIN1*:*GFP S1*,*2*,*3E pin1* (Figure 3I) but only

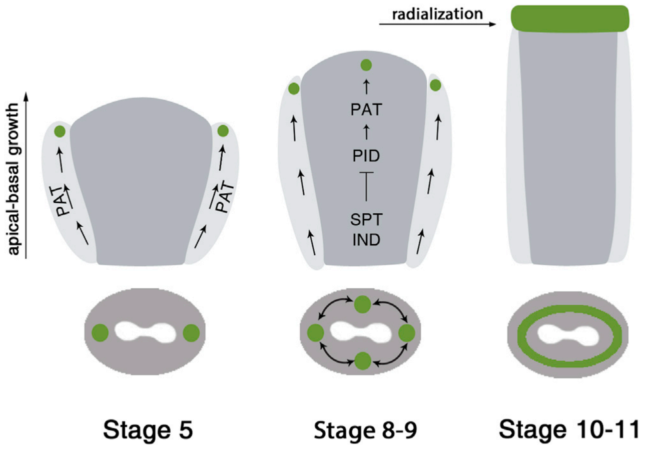

Figure 4. Model for Radiality Establishment at the Top End of a Growing Organ

Model showing how auxin-signaling accumulation (green) through polar auxin transport (PAT, arrows) presides over the bilateral-to-radial symmetry switch during gynoecium development. At stage 5, auxin signaling peaks at the lateral top part of the gynoecium, sustaining the apical-basal growth. At stages 8 and 9, SPT and IND repress *PID* expression, thus promoting apolar PIN localization leading to accumulation of auxin signaling at the medial top and subsequently formation of the radial auxin ring at stages 10 and 11.

in the lateral foci, thereby resembling *DR5* distribution in *spt* and *ind spt* mutants (Figures 2O, 2R, and S2H–S2J).

These results suggest that PID-mediated phosphorylation of PIN1 is sufficient to prevent radial symmetry. As expected, loss of PIN1 phosphorylation had no effect on radial symmetry establishment, because constitutive apolar localization of the PIN1:GFP S1,3A mutant protein sustains apolar auxin flux (Figures 3G, 3H, S3A, and S3B). Together, these results show that apolar localization of PIN1 is required for radial style formation.

Lateral Auxin Foci Are Required for the Medial Auxin Foci to Promote Radial Symmetry

We next tested the developmental relevance of the sequential appearance of the lateral and medial pairs of foci. The gynoecium phenotype resulting from crosses between *pid* loss-of-function mutants and *spt-12* was analyzed to distinguish between two possible scenarios: (1) if activity of the medial foci is sufficient for radial symmetry establishment, complementation of the *spt* split-style phenotype was expected by eliminating PID function and (2) if the role of lateral foci is functionally upstream of the medial foci, a failure to establish radial style development was expected in the double mutant. Analysis of the *pid-8 spt-12* and *pid-9 spt-12* double mutants revealed a strong enhancement of the *spt-12* phenotype and a complete failure in radial symmetry establishment. This result is in agreement with the second scenario and suggests that the lateral foci are required to support the role of the medial foci during style development (Figures 3J, S3E, and S3F).

To study whether the split-style phenotype in *spt* gynoecia is due to a failure of SPT in controlling auxin transport in the medial apex, we introgressed the *PIN1::PIN1:GFP S1,3A* loss-of-phosphorylation mutant into *spt-12*. Here, the background was kept wild-type for the endogenous *PIN1* gene to sustain formation of the lateral foci and promote apical-basal growth. Gynoecia from this genetic combination exhibited complete restoration of the split defect with perfectly radialized styles (Figures 3K, S3C, and S3G). This was dependent on wild-type endogenous PIN1 in the background, because gynoecia from the *PIN1::PIN1:GFP S1,3A spt-12 pin1* triple combination phenocopied *spt pid* double mutant gynoecia (Figures 3J and 3L). As with the *spt pid* double mutants, this triple combination was unable to sustain the apical-basal growth, thus affecting the activity of the lateral foci and enhancing the *spt* phenotype (Figures 3L, S3D, and S3H).

Overall, these results show that SPT (and IND) controls radiality at the gynoecium apex by controlling auxin transport, thus governing auxin flux in the medial region of the style. They also reveal that activity of the medial foci is linked to and dependent on the lateral auxin-signaling foci.

The functional relation between the lateral and medial auxin-signaling foci described here is closely aligned with the stereotypical stages occurring during gynoecium development. As indicated in Figure 4, the early function of the lateral foci is to sustain apical-basal growth allowing to build up the ovary. Subsequently, at stages 8 and 9, in order to obtain a radialized apical style, SPT and IND establish the medial foci by directly repressing *PID* expression [15, 16], thus sustaining apolar PIN localization and auxin accumulation (Figure 4). It is unknown what stimulates expression of the *IND/SPT* module, but it is an intriguing possibility that a feedback mechanism exists between IND/SPT and auxin. Finally, we hypothesize that a long-distance signal is required to connect the different foci in a radial auxin-signaling maximum to achieve a switch in cell polarity and thus orchestrating the coordinated growth of the radial style to facilitate fertilization.

Conclusions

Excellent progress has been made in understanding how auxin provides polarity and identity to cells in a range of developmental contexts. The example presented here demonstrates that auxin can also be recruited to coordinate a heterogeneous group of cells to commit to a program, which imposes homogeneous identity to them. This activity leads to an unusual developmental bilateral-to-radial symmetry transition in the *Arabidopsis* style.

The radial style is a general feature of the female reproductive organ in angiosperms, which arose during the Cretaceous period 100–125 million years ago. The early angiosperms underwent a remarkably rapid diversification and have since reached ecological domination in the plant kingdom in terms of number of species (>300,000) [31]—a phenomenon that Charles Darwin referred to as "the abominable mystery" [32]. Because a radial style is necessary to facilitate efficient fertilization, radialization of the style may have been a key event in allowing the success of flowering plants.

Supplemental Information

Supplemental Information includes Supplemental Experimental Procedures, three figures, and one movie and can be found with this article online at http://dx.doi.org/10.1016/j.cub.2014.09.080.

Author Contributions

L.M. and L.Ø. conceived the hypothesis and planned the experiments, L.M. carried out the experimental work, and L.M. and L.Ø. analyzed the data and wrote the manuscript.

Acknowledgments

We thank Samantha Fox and JIC Bioimaging facility for technical support and Remko Offringa, Jiří Friml, Michael Lenhard, and Jose M. Alonso for

seeds. We are grateful for discussions and helpful comments on the manuscript from Marie Brüser, Joyita Deb, Łukasz Łangowski, Xinran Li, Remko Offringa, Nicola Stacey, Pauline Stephenson, and Eva Sundberg. This work was supported by grant BB/K008617/1 to L.Ø. from the Biotechnological and Biological Sciences Research Council and by the Institute Strategic Programme grant (BB/J004553/1) to the John Innes Centre.

Received: August 26, 2014
Revised: September 29, 2014
Accepted: September 29, 2014
Published: November 6, 2014

References

1. Gerhart, J. (2004). Symmetry breaking in the egg of *Xenopus laevis*. In Gastrulation: From Cells to Embryos, C.D. Stern, ed. (Cold Spring Harbor: Cold Spring Harbor Laboratory Press), pp. 341–351.
2. Kimelman, D., and Bjornson, C. (2004). Vertebrate mesoderm induction: from frogs to mice. In Gastrulation: From Cells to Embryos, C.D. Stern, ed. (Cold Spring Harbor: Cold Spring Harbor Laboratory Press), pp. 363–372.
3. Akiyama-Oda, Y., and Oda, H. (2006). Axis specification in the spider embryo: dpp is required for radial-to-axial symmetry transformation and sog for ventral patterning. Development *133*, 2347–2357.
4. Aida, M., Vernoux, T., Furutani, M., Traas, J., and Tasaka, M. (2002). Roles of PIN-FORMED1 and MONOPTEROS in pattern formation of the apical region of the Arabidopsis embryo. Development *129*, 3965–3974.
5. Collins, A.G., and Valentine, J.W. (2001). Defining phyla: evolutionary pathways to metazoan body plans. Evol. Dev. *3*, 432–442.
6. Holley, S.A., Jackson, P.D., Sasai, Y., Lu, B., De Robertis, E.M., Hoffmann, F.M., and Ferguson, E.L. (1995). A conserved system for dorsal-ventral patterning in insects and vertebrates involving sog and chordin. Nature *376*, 249–253.
7. Lowe, C.J., and Wray, G.A. (1997). Radical alterations in the roles of homeobox genes during echinoderm evolution. Nature *389*, 718–721.
8. Pawson, D.L. (2007). Phylum echinodermata. Zootaxa *1668*, 749–764.
9. Liljegren, S.J., Roeder, A.H., Kempin, S.A., Gremski, K., Østergaard, L., Guimil, S., Reyes, D.K., and Yanofsky, M.F. (2004). Control of fruit patterning in *Arabidopsis* by INDEHISCENT. Cell *116*, 843–853.
10. Heisler, M.G., Atkinson, A., Bylstra, Y.H., Walsh, R., and Smyth, D.R. (2001). *SPATULA*, a gene that controls development of carpel margin tissues in *Arabidopsis*, encodes a bHLH protein. Development *128*, 1089–1098.
11. Roeder, A.H.K., and Yanofsky, M.F. (2006). Fruit development in *Arabidopsis*. Arabidopsis Book *4*, e0075.
12. Scutt, C.P., Vinauger-Douard, M., Fourquin, C., Finet, C., and Dumas, C. (2006). An evolutionary perspective on the regulation of carpel development. J. Exp. Bot. *57*, 2143–2152.
13. Anastasiou, E., Kenz, S., Gerstung, M., MacLean, D., Timmer, J., Fleck, C., and Lenhard, M. (2007). Control of plant organ size by KLUH/CYP78A5-dependent intercellular signaling. Dev. Cell *13*, 843–856.
14. Smyth, D.R., Bowman, J.L., and Meyerowitz, E.M. (1990). Early flower development in *Arabidopsis*. Plant Cell *2*, 755–767.
15. Girin, T., Paicu, T., Stephenson, P., Fuentes, S., Körner, E., O'Brien, M., Sorefan, K., Wood, T.A., Balanzá, V., Ferrándiz, C., et al. (2011). INDEHISCENT and SPATULA interact to specify carpel and valve margin tissue and thus promote seed dispersal in *Arabidopsis*. Plant Cell *23*, 3641–3653.
16. Sorefan, K., Girin, T., Liljegren, S.J., Ljung, K., Robles, P., Galván-Ampudia, C.S., Offringa, R., Friml, J., Yanofsky, M.F., and Østergaard, L. (2009). A regulated auxin minimum is required for seed dispersal in Arabidopsis. Nature *459*, 583–586.
17. Larsson, E., Franks, R.G., and Sundberg, E. (2013). Auxin and the Arabidopsis thaliana gynoecium. J. Exp. Bot. *64*, 2619–2627.
18. Stepanova, A.N., Robertson-Hoyt, J., Yun, J., Benavente, L.M., Xie, D.Y., Dolezal, K., Schlereth, A., Jürgens, G., and Alonso, J.M. (2008). TAA1-mediated auxin biosynthesis is essential for hormone crosstalk and plant development. Cell *133*, 177–191.
19. Gälweiler, L., Guan, C., Müller, A., Wisman, E., Mendgen, K., Yephremov, A., and Palme, K. (1998). Regulation of polar auxin transport by AtPIN1 in Arabidopsis vascular tissue. Science *282*, 2226–2230.
20. Petrásek, J., Mravec, J., Bouchard, R., Blakeslee, J.J., Abas, M., Seifertová, D., Wisniewska, J., Tadele, Z., Kubes, M., Covanová, M., et al. (2006). PIN proteins perform a rate-limiting function in cellular auxin efflux. Science *312*, 914–918.
21. Benjamins, R., Quint, A., Weijers, D., Hooykaas, P., and Offringa, R. (2001). The PINOID protein kinase regulates organ development in *Arabidopsis* by enhancing polar auxin transport. Development *128*, 4057–4067.
22. Christensen, S.K., Dagenais, N., Chory, J., and Weigel, D. (2000). Regulation of auxin response by the protein kinase PINOID. Cell *100*, 469–478.
23. Friml, J., Yang, X., Michniewicz, M., Weijers, D., Quint, A., Tietz, O., Benjamins, R., Ouwerkerk, P.B., Ljung, K., Sandberg, G., et al. (2004). A PINOID-dependent binary switch in apical-basal PIN polar targeting directs auxin efflux. Science *306*, 862–865.
24. Huang, F., Zago, M.K., Abas, L., van Marion, A., Galván-Ampudia, C.S., and Offringa, R. (2010). Phosphorylation of conserved PIN motifs directs *Arabidopsis* PIN1 polarity and auxin transport. Plant Cell *22*, 1129–1142.
25. Bennett, S.R.M., Alvarez, J., Bossinger, G., and Smyth, D.R. (1995). Morphogenesis in *pinoid* mutants of *Arabidopsis thaliana*. Plant J. *8*, 505–520.
26. Nemhauser, J.L., Feldman, L.J., and Zambryski, P.C. (2000). Auxin and *ETTIN* in *Arabidopsis* gynoecium morphogenesis. Development *127*, 3877–3888.
27. Ståldal, V., Sohlberg, J.J., Eklund, D.M., Ljung, K., and Sundberg, E. (2008). Auxin can act independently of CRC, LUG, SEU, SPT and STY1 in style development but not apical-basal patterning of the Arabidopsis gynoecium. New Phytol. *180*, 798–808.
28. Blilou, I., Xu, J., Wildwater, M., Willemsen, V., Paponov, I., Friml, J., Heidstra, R., Aida, M., Palme, K., and Scheres, B. (2005). The PIN auxin efflux facilitator network controls growth and patterning in Arabidopsis roots. Nature *433*, 39–44.
29. Friml, J., Benková, E., Blilou, I., Wisniewska, J., Hamann, T., Ljung, K., Woody, S., Sandberg, G., Scheres, B., Jürgens, G., and Palme, K. (2002). AtPIN4 mediates sink-driven auxin gradients and root patterning in Arabidopsis. Cell *108*, 661–673.
30. Petrásek, J., and Friml, J. (2009). Auxin transport routes in plant development. Development *136*, 2675–2688.
31. Ferrándiz, C., Fourquin, C., Prunet, N., Scutt, C.P., Sundberg, E., Trehin, C., and Vialette-Guiraud, A.C.M. (2010). Carpel development. In Advances in Botanical Research, J.-C. Kader and M. Delseny, eds. (Burlington: Academic Press), pp. 1–73.
32. Friedman, W.E. (2009). The meaning of Darwin's 'abominable mystery'. Am. J. Bot. *96*, 5–21.

Current Biology, Volume *24*

Supplemental Information

Dynamic Control of Auxin Distribution Imposes a Bilateral-to-Radial Symmetry Switch during Gynoecium Development

Laila Moubayidin and Lars Østergaard

Supplemental Figures

Figure S1.

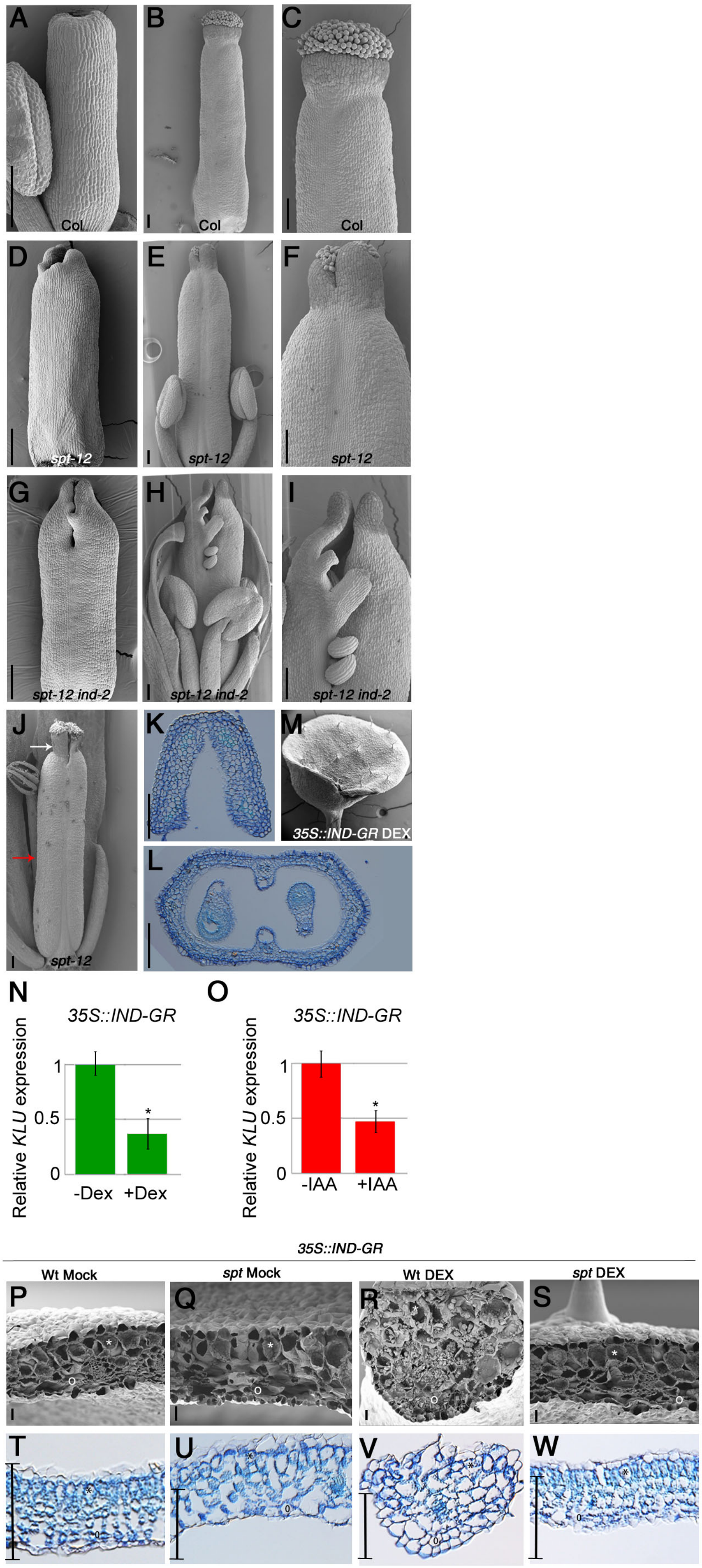

Figure S1. SPT and IND activities are both necessary and sufficient to govern organ radiality, Related to Figure 1.

(A-C) SEM images of gynoecia from Col-0 at stages 8 (A) and 10 (B,C). (D-F) SEM images of gynoecia from *spt-12* at stages 8 (D) and 10 (E,F). (G-I) SEM images of gynoecia from *ind-2 spt-12* at stages 8 (G) and 10 (H,I). (J) SEM of *spt-12* gynoecium at stage 12. White arrow indicates the style region, red arrow indicates the ovary. (K,L) Toluidine blue-staining of cross sections from *spt-12* stage-12 gynoecium in the style (K) and ovary (L). (M) SEM image of radicalized, cup-shaped seedling from *35S::IND:GR* on 10µM DEX. Scale bars in (A-M) represent 100µm.

(N) qRT-PCR of *KLU* in *35S::IND:GR* with 10µM DEX. (O) qRT-PCR of *KLU* with 50µM IAA. Error bars show SD. Student‘s t-test, *$p<0.05$. (P-W) SEM images (P-S) and toluidine blue-stained (T-W) of sections of rosette leaf from *35S::IND:GR* in Col-0 (P,T) and *spt-12* (Q,U) without DEX and *35S::IND:GR* in Col-0 (R,V) and *spt-12* (S,W) with 10µM DEX. Asterisks indicate the palisade parenchyma, circles indicate sponge parenchyma. Note that radialized leaves emerging after IND overexpression appear not to show changing in the identity of the canonical internal leaf tissues. Scale bars in (P-S) represent 10µm, in (Q-T) represent 100µm.

Figure S2.

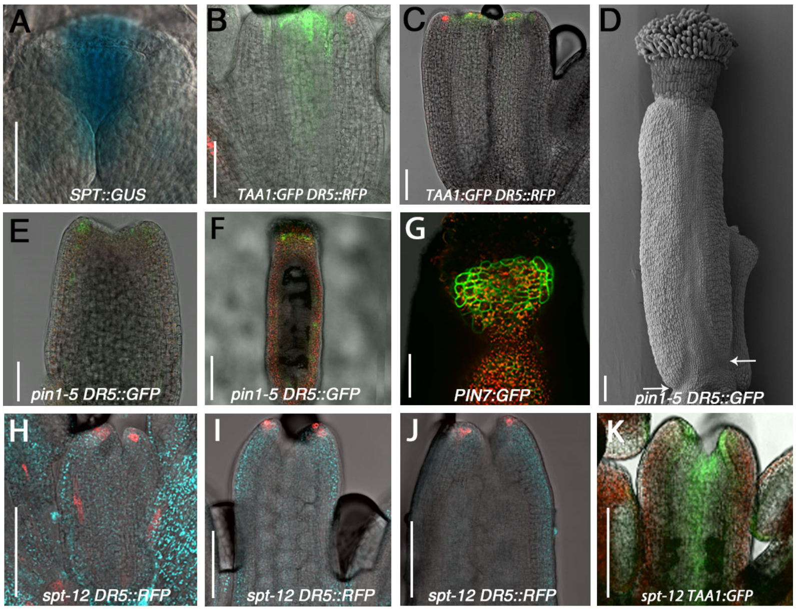

Figure S2. Lateral and medial auxin-signaling foci drive apical-basal growth and style formation, respectively, during gynoecium development, Related to Figure 2.

(A) *SPT::GUS* in the medial region of a stage-5 Col-0 gynoecium. Scale bar represents 50µm. (B-C) Confocal image of *TAA1::TAA1:GFP* and *DR5::RFP* of a stage-6 (B) and a stage-9 (C) Col-0 gynoecium. *TAA1::TAA1:GFP* and *DR5::RFP* are expressed in complementary regions during early developmental stages since *TAA1::TAA1:GF*P expression is constrained in the medial region while *DR5::RFP* shows expression in the lateral (B). At stage-9 *TAA1::TAA1:GFP* and *DR5::RFP* expression overlaps since *TAA1::TAA1:GFP* starts to be expressed in the top adaxial side of the lateral region while *DR5::RFP* is expressed in the top medial foci. Scale bar represents 50µm. (D-F) SEM image (D) and confocal analysis (E,F) of *pin1-5 DR5::GFP* gynoecium at stage 11 (D), stage 7 (E) and 10 (F). White arrows in (C) indicate the base of the ovary. Scale bars represent in (D) 100µm and in (E,F) 50µm. (G) *PIN7::PIN7:GFP* in stage-10 Col-0 gynoecium. Scale bar

represents 50μm. (H-J) *DR5::RFP* in *spt-12* at stages 5-6 (F), 8-9 (G) and 10 (H). Note the absence of the medial *DR5* signaling foci over developmental stages. Scale bars represent 100μm. (K) *TAA1::TAA1:GFP* expression in *spt-12* at stage 5-6. Scale bar represents 100 μm.

Figure S3.

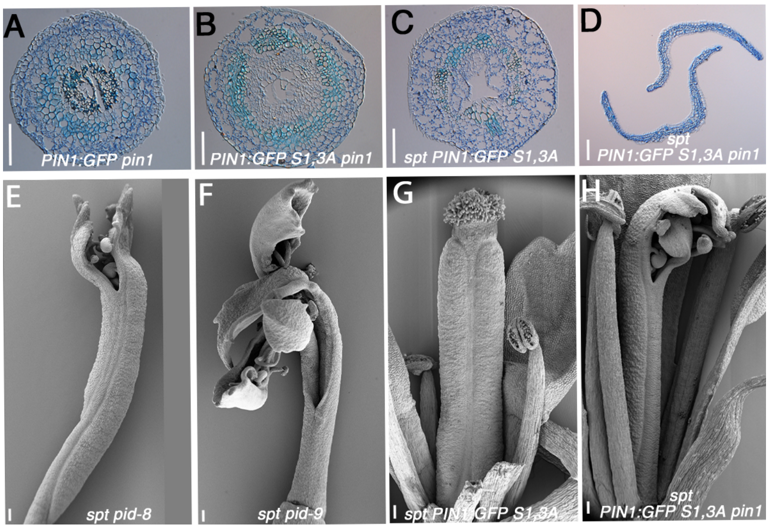

Figure S3. Radial style is formed by the coordinated activity of lateral and medial auxin foci, Related to Figure 3.

(A-D) Toluidine blue-stained style sections in *PIN1::PIN1:GFP pin1* (A), *PIN1::PIN1:GFP S1,3A pin1* (B), *PIN1::PIN1:GFP S1,3A spt-12* (C) and *PIN1::PIN1:GFP S1,3A spt-12 pin1* (D). Scale bars represent 100μm. (E-H) SEM images of stage-10 gynoecia from *pid-8 spt-12* (E), *pid-9 spt-12* (F), *PIN1::PIN1:GFP S1,3A spt-12* (G), and *PIN1::PIN1:GFP S1,3A spt-12 pin1* (H). Scale bars in (A-D) represent 100μm.

Supplemental Experimental Procedures

Plant Materials and Growth Conditions

Plants were grown on soil in long days condition (16h light/8h dark) in the glasshouse. Mutant lines *ind-2 spt-12* [15], *spt-12* [15,S1], *35S::IND-GR* and *35S::IND-GR spt-12* [15,16], *pid-9* [20], *PIN1::PIN1:GFP pin1*, *PIN1::PIN1:GFP S1,3A pin1* and *PIN1::PIN1:GFP S1,2,3E pin1* [22] were in Col-0 background. *pid-8* [23] was in Ws background. Reporter lines of *SPT::GUS* [14,S2], *KLU::GUS* [11], *PID::GUS* [19], *TAA1::TAA1:GFP* [17], *DR5::GFP* [S3], *DR5::RFP* [S4], *pin1 DR5::GFP* [S5], *PIN1::PIN1:GFP* [S6], *PIN3::PIN3:GFP* [S7], *PIN7::PIN7:GFP* [28].

RNA extraction and qRT-PCR

Total RNA was isolated from Col-0, *spt-12* and *spt-12 ind-2* inflorescences and from *35S::IND-GR* seedlings treated or untreated with DEX or IAA as shown in [16], and treated with RNeasy Plant Mini Kit (QIAGEN). Each RNA sample was reverse transcribed using the M-MLV Reverse Transcriptase (Promega) according to the manufacturer's instructions. qRT-PCR was performed in triplicates from each RNA sample and repeated twice using BRYT Green based GoTaq qPCR Master Mix (Promega) with Chromo4 Real-Time PCR Detection System (Bio-Rad). Expression levels were calculated relative to *UBIQUITIN 10* using the $2^{-\Delta\Delta ct}$ method. Statistical analysis was done in MS Exel (ANOVA: Single Factor) using $p<0.05$. Primers were designed according to the recommendations of Applied Biosystems Quantitative RT-PCR (qRT-PCR) analysis was conducted using the gene-specific primers listed below:

for *KLU*:

KLU FWD: AGGCTGGTGAGTGAAGGCTA

KLU REV: CAAGCCAAGCAAGACATCAA

for *UBIQUITIN 10*:

UB10 FWD: AGAACTCTTGCTGACTACAATATCCAG

UB10 REV: GTTAAGACGTTGACTGGGAAAACTAT

GUS histochemical assay

To visualize *KLU::GUS*, *PID::GUS* and *SPT::GUS* lines, GUS histochemical assay was performed using 1 mg/ml of ß-glucoronidase substrate X-gluc (5-bromo-4-chloro-3-indolyl glucuronide, MELFORD) dissolved in Dimethyl sulfoxide (DMSO). X-Gluc solution contains 100 mM sodium phosphate buffer, 10mM EDTA, 0.5 mM K_3 $Fe(CN)_6$, 3 mM $K_4Fe(CN)_6$, 0.1% Triton X100 according to the JIC standard operating procedures. Wild type and mutant inflorescences of *KLU::GUS* and *PID::GUS* were vacuum infiltrated for 10' and incubated for 16 hours at 37°C in the dark. *SPT::GUS* inflorescences were pre-treated for 1h with acetone at -20°C, washed two times for 5' in 100 mM sodium phosphate buffer, washed for 30' in 100 mM sodium phosphate buffer containing 1mM K_3 - K_4 at room temperature and then incubated for 2h at 37°C in the X-Gluc solution. After staining, the reaction buffer was replaced with 70% ethanol until chlorophyll was completely washed out from the samples. Gynoecium were dissected, mounted in Chlorohydrate (Sigma) solution and analyzed using Leica DM600 light microscopy. Images were taken using Leica LAS AF7000 software.

Toluidine Blue Staining

Tissues were fixed for 16h at 25°C in 3.7% formaldehyde, 5% acetic acid, and 50% ethanol and subsequently dehydrated through an ethanol series until 70%. The tissues were embedded in paraffin. An RM 2125 rotary microtome (Leica) was used to make 10 mm transverse sections of Col, *spt-12* and *spt-12 ind-2* gynoecium at stage 12, and *35S::IND-GR* and *spt-12 35S::IND-GR* leaves treated with either mock or DEX. Sections were deparaffinizated by two rounds of incubation in 100% Histoclear (National Diagnostics) for 10' at room temperature followed by two washes in 100% ethanol for 2' at room temperature, air dried for 30' and stained for 10' by an aqueous solution containing 0.005% Toluidine blue O (ACROS ORGANICS). Slides were washed for 1' in water; sections were mounted in a histological mounting medium Histomount (National Diagnostic) and examined under Leica DM600 light microscopy. Images were taken using Leica LAS AF7000 software.

Scanning Electron Microscopy

Different stages of Col, *spt-12* and *spt-12 ind-2* gynoecium and seedlings of *35S::IND-GR* and *spt-12 35S::IND-GR* leaves treated with either mock or DEX were fixed 16h at 25°C in 3.7% formaldehyde, 5% glacial acetic acid, and 50% ethanol. After a complete dehydration through an ethanol series until 100%, gynoecium and leaves were critical point dried. For the sections of *35S::IND-GR* and *spt-12 35S::IND-GR* leaves in Figure 1 and S1, only the first leaves were used and cut before the critical point drying. Samples were dissected and coated with gold and examined under Zeiss Supra 55VP Field Emission Scanning Electron Microscope using an acceleration voltage of 3 kV.

DEX and IAA inductions

For *IND* ectopic induction, seeds carrying the *35S::IND-GR* construct, in wild type and in *spt-12* background, were surface sterilized using 50% bleach for 10 minutes and then rinsed four times with sterile water. After 5 days of cold treatment, *A. thaliana* seeds were plated and grown for two weeks, in a horizontal position, at 22°C in long-day conditions (16 hours light/8 hours dark cycle) on MS (Murashige & Skoog) medium containing micro and macro elements including vitamins (Formedium Limited), 3% sucrose at pH 5.8, supplemented with 10 µM dexamethasone (Dex, Sigma-Aldrich), prepared from a 10 mM stock in ethanol, or an equivalent amount of ethanol, as mock treatment. For qRT-PCR experiment with *35S::IND-GR* construct, seedlings were treated as previously shown in [16].

Confocal Microscopy

Confocal microscopy was performed using a Leica SP5 laser scanning microscope equipped with an Argon krypton laser (Leica Microsystems). The 488-nm and 561-nm excitation line of an argon ion laser was used to excite GFP and RFP, respectively. GFP emission spectra were collected between 497 and 551 nm, RFP emission spectra were collected between 570 and 630 nm, and plastid autofluorescence was collected between 624 and 699nm. For the lateral view of gynoecium in Figures 2A, 2C, 2E, 2G, 2I, 2J, 2M-2R and S2B, S2C, S2E-S2J

floral buds were dissected, mounted in water and observed using transmitted light (bright field) using x10 air or x40 oil objectives. For the top views of style region in Figures 2B,D,F,H,K,L and 3D,E,G gynoecium were dissected and mounted vertically in an agars dishes, we used the X25/0.95 water dipping objective lens that allowed a three dimensional visualization of the specimens. Images were averaged 8 times and were processed using the Leica CONFOCAL software. For Figures 2M-2O, 3I and Movie1, 2-3μm Z sections were imaged and, for the figures, converted to 3D projections.

Supplemental References

[S1] Ichihashi, Y., Horiguchi, G., Gleissberg, S., and Tsukaya, H. (2010). The bHLH transcription factor SPATULA controls final leaf size in *Arabidopsis thaliana*. Plant Cell Physiol *51*, 252-261.

[S2] Groszmann, M., Bylstra, Y., Lampugnani, E.R., and Smyth D.R. (2010). Regulation of tissue-specific expression of SPATULA, a bHLH gene involved in carpel development, seedling germination, and lateral organ growth in *Arabidopsis*. J Exp Bot *61*, 1495-1508.

[S3] Ottenschläger, I., Wolff, P., Wolverton, C., Bhalerao, R.P., Sandberg, G., Ishikawa, H., Evans, M., and Palme, K. (2003). Gravity-regulated differential auxin transport from columella to lateral root cap cells. Proc. Natl Acad. Sci. USA *100*, 2987–2991.

[S4] Marin, E., Jouannet, V., Herz, A., Lokerse, A.S., Weijers, D., Vaucheret, H., Nussaume, L., Crespi, M.D., and Maizel, A. (2010). miR390, Arabidopsis TAS3 tasiRNAs, and their AUXIN RESPONSE FACTOR targets define an autoregulatory network quantitatively regulating lateral root growth. Plant Cell *22*, 1104–1117.

[S5] Ružicka, K., Ljung, K., Vanneste, S., Podhorská, R., Beeckman, T., Friml, J. and Benková, E. (2007). Ethylene regulates root growth through effect on auxin biosynthesis and transport-dependent auxin distribution. Plant Cell *19*, 2197-2212.

[S6] Benková, E., Michniewicz,M., Sauer, M., Teichmann, T., Seifertová D., Jurgens, J., and Friml, J. (2003). Local, Efflux-Dependent Auxin Gradients as a Common Module for Plant Organ Formation. Cell *115*, 591-602.

[S7] Žádníková, P., Petrášek, J., Marhavý, P., Raz, V., Vandenbussche, F, Ding, Z., Schwarzerová, K., Morita, M.T., Tasaka, M., Hejátko, J., Van Der Straeten, D., Friml, J., and Benková, E. (2010). Role of PIN-mediated auxin efflux in apical hook development of Arabidopsis thaliana. Development *137*, 607-617.
